# An oral history of medical laboratory development in francophone West African countries

**DOI:** 10.4102/ajlm.v10i1.1157

**Published:** 2021-03-16

**Authors:** Winny Koster, Albert G. Ndione, Mourfou Adama, Ibrehima Guindo, Iyane Sow, Souleymane Diallo, Jean Sakandé, Pascale Ondoa

**Affiliations:** 1 Amsterdam Institute for Global Health and Development, Amsterdam, the Netherlands; 2 Department of Public Health, Faculty of Social and Behavioural Sciences, Amsterdam Institute for Social Science Research, University of Amsterdam, Amsterdam, the Netherlands; 3 Institut de Recherche pour le Développement (IRD), TransVIHMI, CRCF-Hôpital Fann, Dakar, Senegal; 4 Centre National de Lutte Anti Tuberculeuse (CNLAT), Laboratoire National de Référence des Mycobactéries, Ouagadougou, Burkina Faso; 5 Institut National de Recherche en Santé Publique, Bamako, Mali; 6 Directorate of Laboratory services, Ministry of Public Health and Social Welfare, Dakar, Senegal; 7 Centre d’Infectiologie Charles Mérieux, Bamako, Mali; 8 Fondation Mérieux, Ouagadougou, Burkina Faso; 9 Department of Global Health, Amsterdam University Medical Centers, Amsterdam, the Netherlands; 10 African Society for Laboratory Medicine, Addis Ababa, Ethiopia

**Keywords:** oral history, medical laboratory, Senegal, Burkina Faso, Mali

## Abstract

**Background:**

Underdeveloped and underused medical laboratories in sub-Saharan Africa negatively affect the diagnosis and appropriate treatment of ailments.

**Objective:**

We identified political, disease-related and socio-economic factors that have shaped the laboratory sector in Senegal, Mali and Burkina Faso to inform laboratory-strengthening programmes.

**Methods:**

We searched peer-reviewed and grey literature from February 2015 to December 2018 on laboratory and health systems development from colonial times to the present and conducted in-depth interviews with 73 key informants involved in (inter)national health or laboratory policy, organisation, practice or training. This article depended on the key informants’ accounts due to the paucity of literature on laboratory development in francophone West African countries. Literature and interview findings were triangulated and are presented chronologically.

**Results:**

Until around 1990 there were a few disease-specific research laboratories; only the larger hospitals and district health facilities housed a rudimentary laboratory. The 1990s brought the advent of donor-dictated, vertical, endemic and epidemic disease programmes and laboratories. Despite decentralising from the national level to the regional and district levels, these vertical laboratory programmes biased national health resource allocation deleteriously neglecting the development of the horizontal, general-health laboratory. After the year 2000, the general-health laboratory system received more attention when, influenced by the World Health Organization, national networks and (sub-)directorates of laboratories were installed.

**Conclusion:**

To advance national general healthcare, as opposed to disease-specific healthcare, national laboratory directors and experts in general laboratory development should be consulted when national policies are made with potential laboratory donors.

## Introduction

Underdeveloped and underused medical laboratories in sub-Saharan Africa hamper the diagnosis and management of potentially epidemic infectious diseases as well as general public health conditions.^1,2,3,4^ Recent national surveys of laboratory capacity conducted in Senegal, Mali and Burkina Faso – francophone West Africa for short, and the countries this study focuses on – found that many laboratories still have insufficient personnel, a lack of or dysfunctional basic equipment, and operate in inappropriate rooms.^5,6,7,8^ Studies in Senegal on barriers to the uptake of the seven recommended routine maternal diagnostic tests found that laboratory problems were part of the reason why only around one-third of pregnant women received the complete set of tests.^9,10^

Identifying the historical developments that have shaped the laboratory sector could help to understand the gaps and provide lessons for laboratory-strengthening programmes. As far as we know, this is the first study on this topic. The guiding question of our study was: what political, socio-economic, and disease-related factors have influenced the current status of medical laboratories in Senegal, Mali and Burkina Faso?

The development of medical laboratories in Africa cannot be dissociated from the development of the healthcare sector at large.^11,12,13,14^ The healthcare sector in colonial times was mainly directed controlling infectious disease control and keeping the colonial workforce healthy to ensure economic productivity.^15^ The years after independence saw optimism towards national economic growth that focused on improved healthcare for the general population.^15^ The global economic recession of the early 1980s and the structural adjustment programmes of the late 1980s–1990s had a significant negative impact on national economies and forced governments to cut spending and lower their development goals of early post-independence.^15,16,17^ The ensuing cuts in the health sector thus created institutional, technological and personnel capacity problems.^13,17,18^ The late 1990s saw the emergence of global health initiatives in response to worldwide health-related problems, mainly epidemic diseases, that could pose global security threats.

Our article progresses chronologically: it begins in late colonial times, goes on to the post-independence period, followed by the time of structural adjustment, and ends with the emergence of global health. In each period, we describe the developments in the laboratory sector and the influencing factors.

## Methodology

### Ethical considerations

The objectives of the study were explained to all key informants; all gave verbal consent to be interviewed and recorded, as can be proven by the audio recordings.

### Study design

The data for this qualitative descriptive study are derived primarily from in-depth key informant interviews and a literature review. The study proposal identified themes in the interrelated factors influencing laboratory development. Themes explored were: organisation of services, regulations, health policies and plans; economic conditions and by extension personnel, equipment, training and infrastructure development; endemic and epidemic disease contexts; and lastly, funding by national governments and (inter)national donors.

### Data collection methods and study participants

#### Literature review

Between February 2015 and February 2016, three country teams of three to six social science researchers and laboratory professionals searched for peer-reviewed and grey literature on the study’s themes and identified factors in local libraries, organisations and government departments (Online Supplementary Document – Table 1), and freely accessible websites (Online Supplementary Document – List 1).

#### Key informant interviews

Key informants, selected by the country team members, were in 2015 interviewed for 30 min to 3 h, mainly at their workplace. Selection criteria were having an active role in the (inter)national health or laboratory policy, organisation, practice or training (past and present). For every key informant, a personalised question guide was prepared, considering their professional background, the period of their professional work and their involvement with laboratories.

A summary of the 73 key informants’ background characteristics is presented in Table 1, details are provided in the Online Supplementary Document – Table 2. Key informants were trained in clinical or laboratory medicine (or both) and included retired (*n* = 8) and current (*n* = 65) staff members of various departments in the Ministry of Health (MoH), international organisations, private and public health facilities, professional associations, training institutions, and universities; many had worked in several functions. One key informant’s professional career commenced during the colonial period, nine commenced in the two decades after the three countries’ independence in 1960, while the remainder commenced from the 1980s.

**TABLE 1:** Background of the 73 key informants, francophone Africa, February 2015 – February 2016.

Variable	*n*	%
**Sex**		
Male	49	67
Female	24	33
**Country**		
Senegal	20	27
Burkina Faso	24	33
Mali	29	40
**Professional training (most recent)**		
Laboratory (assistant, technician, pharmacist, biologist)	40	55
Clinical (nurse, midwife, medical doctor, gynaecologist)	32	44
Other	1	1
**Period interviewee started work**		
Colonial	1	1
1960–1979	9	12
1980–1999	48	66
2000–2014	15	21
**Present or last workplace before retirement (multiple response)**		
At training institute (university, training school for laboratory technicians, midwives)	19	26
At Ministry of Health (division or [sub-] directorate laboratories, other directorates)	16	22
Private practice (laboratory, maternity)	12	16
Laboratory practice	10	14
Clinical practice	9	12
Research institute	7	9
International organisation	6	8
Retired	8	11

### Data analysis and presentation

The audio-recorded key informant interviews (in French) were transcribed verbatim. NVivo qualitative data analysis software, version 10 (QSR International, Melbourne, Australia), was used for the thematic analysis of the interviews and a timeline was constructed for each country. Each country team summarised their literature findings thematically and chronologically. In this article, the findings derived from the two data collection methods have been triangulated and are presented for the three countries combined. Occasionally, differences and specificities across countries are pointed out. It should be noted that when the article refers to literature, the same information was usually corroborated by key informants. All quotations in the text are English translations of the French citations.

## Results

The study’s primary finding and challenge was the scarcity of written sources on laboratories; consequently, this history of laboratory development mainly hinges on the accounts of key informants, all of whom willingly recounted their experiences. Notably, those involved in laboratory practice or policy welcomed the attention; as a Senegalese biologist involved in laboratories since 1982 expressed: ‘The laboratory has habitually been the forgotten part of medicine, meaning one thinks of everything, and of the laboratory only after that’ (Key informant 62, male, Interview date 11 March 2015, Dakar). Descriptions of developments for colonial times and during the first decades after independence are briefer than for the periods afterwards because most of the key informants commenced their laboratory experience after these two periods.

### Colonial times

In colonial times, all three countries housed renowned research laboratories, including *Centre Muraz* in Burkina Faso, *Institut Pasteur* in Senegal and *Laboratoire Central de Biologie* in Mali. These laboratories were involved in the research and the control of endemic and epidemic diseases – including trypanosomiasis, cholera, onchocerciasis, meningitis, malaria and syphilis – to preserve the labour and military force,^15^ and were funded by FIDES (*Fonds d’Investissements pour le Développement Economique et Social des territoires d’outre-mer*).^19^ The staff were French doctors and local assistants – mainly nurses – trained on-the-job.^19,20^ A retired Burkinabé pharmacist stated that everything related to laboratories in French West Africa started with colonial doctors interested in tropical diseases; for instance, Gaston Muraz, a French military doctor, created *Centre Muraz* in Bobo Dioulasso in 1939. The few existing national and regional hospitals had small laboratories attached.^19^ In Dakar, the capital of French West Africa – which included present-day Burkina Faso and Mali – Principal, Dantec and Fann hospitals were among the first modern healthcare facilities in sub-Saharan Africa.^21^ Mali had Kayes, Mopti, Markala and Point G hospitals. In Burkina Faso, the first hospital was a military facility at Bobo Dioulasso (now the University Hospital Sanou Sourô). A retired director of *Institut National de Recherche en Santé Publique* (INRSP) in Mali, who replaced the French director of the Central Laboratory in Bamako in 1956, remembered that some pharmacies also had a small laboratory attached.

### Post-independence (≈1960–1979)

After independence, the colonial research laboratories remained – although some under another name (the Malian *Laboratoire Central de Biologie* became *Institut National de Biologie Humaine*) – as did those attached to the few public national and regional hospitals.^22^ A few medical laboratories were attached to private pharmacies in Burkina Faso, while Mali had 16 public stand-alone medical laboratories, mainly for technical support of disease control programmes.^19^

Laboratory diagnostic tests were limited and aimed mainly at detecting parasites and measuring glycosuria. Even in larger public hospitals, laboratory services were embryonic and only a few laboratory training programmes existed. In Burkina Faso, nurses could specialise in laboratory technology at *Ecole Jamot* and *Centre Muraz*. In Mali, schools were opened for medical doctors and pharmacists, two of which offered biology: *l’École des Assistants Médicaux* (founded 1969) and *l’École Nationale de Médecine et de Pharmacie* (founded 1974). In Mali, a microbiology research laboratory was set up in 1973, where many of the Malian key informants received training. The head of this laboratory had a wide network of international partners who helped provide the necessary resources to make the laboratory internationally renowned. Laboratory assistants were trained in the Secondary School of Health (founded 1963) and the *Institute National de Formation en Sciences de la Santé*. In Senegal, the Faculty of Medicine at Cheikh Anta Diop University (founded 1962) trained pharmacists who worked all over West Africa.^21^ In the three countries, assistants trained on-the-job represented a large part of the laboratory workforce.

The national MoH in Senegal and Burkina Faso did not prioritise laboratories, resulting in a lack of equipment and supplies for the few existing public facilities. In Mali, however, the Minister of Health acknowledged the importance of laboratories relatively early; he created the national research laboratory INRSP in 1973^23^ and aimed to strengthen public hospital laboratories and establish a national coordinating body and policy. The former INRSP director, recounted that in 1974 this health minister created the Division of Laboratories and appointed him head, telling him to assess the status of laboratories in the country. Based on his situation analysis, he recommended that laboratory services be decentralised to the regional level. Disease outbreaks, in particular the 1974–1975 meningitis and cholera outbreaks, added to the health minister’s motivation to decentralise laboratory services. The former INRSP director remembered how cumbersome the control of these outbreaks had been, notably the transportation of suspected cases’ stool samples from the regional laboratories to the central laboratories for analysis.

### Primary healthcare and structural adjustment (≈1980–the late 1990s)

This period saw the implementation of two global strategies for health systems development: the 1978 Alma Ata Primary Health Care Declaration and the 1988 Bamako Initiative. These called for the decentralisation of basic health services, a focus on clinical diagnosis and the supply of essential drugs and equipment. However, structural adjustment programmes, with their efficiency-driven economic reforms, implied less state involvement, public spending cuts and privatisation of healthcare services.^15,17^

#### Decentralisation of laboratories

In the three countries, health centres at (sub-)district level were built, usually with a supporting laboratory to provide the stipulated minimum of primary healthcare diagnostic tests.^24,25^ The revolutionary Sankara regime (1983–1987) aimed to bring healthcare, including rudimentary laboratories, to all corners of Burkina Faso, as the then minister of health and sports (1985–1987) explained. A pharmacist and current health inspector recounted that the reorganisation of the Burkinabé national health system into districts became a fact in 1992–1993, with service norms set by level; laboratories were designated to the district level.

Medical doctors and laboratory personnel working at district level narrated that at this time if there was a laboratory at all, it was rudimentary and very few types of tests were done, usually in a room not designed to house a laboratory. A Burkinabé laboratory technician depicted how, in the 1980s, a typical health centre laboratory was just a small room with a microscope, a manual centrifuge and a fridge with some reagents. ‘Community people called us “stool doctors” because they only saw us examining stools’ (Key informant 35, male, interview date 06 May 2015, Ouagadougou), recounted the lecturer for laboratory technicians at *Ecole Nationale de Santé Publique* in Ouagadougou. A challenge for peripheral laboratories was the absence or unreliability of electricity. A medical doctor, currently at *Conseil National de Lute contre le SIDA*, shared his experiences in rural Burkina Faso:

‘During the years 1984–1990, it was challenging. It was apparent that when you were in a place without electricity you could use very little equipment. Only those working in private laboratories had solar microscopes. Thus, we had to seek a window and work with sunlight; in that way, we could only do parasitology tests’. (Key informant 46, male, interview date 04 May 2015, Ouagadougou)

He concluded: ‘Sophisticated laboratory development followed electrification’. His midwife colleague at *Conseil National de Lute contre le SIDA* added that most health problems were diagnosed at this time through physical patient examination. Two Senegalese medical doctors, both working in district health centres, remembered that in the 1980s and 1990s, they only sporadically referred patients to regional hospital laboratories for diagnostic tests if the patients could afford the travel costs.

Key informants in Mali and Burkina Faso reported that structural adjustment programmes had stimulated private practice; this was supported by government laws authorising private medical practice.^26^ Consequently, some private laboratories were created, either stand-alone, like *Rive Droite* in Bamako, or attached to private hospitals (six in Burkina Faso). This legal opening prompted some pharmacists to leave the public sector and open private pharmacies with a laboratory attached. The Malian and Burkinabé key informants generally applauded the development of private laboratories because it increased access to laboratory services, though they acknowledged quality control challenges.

#### Training opportunities and insufficiencies

Training opportunities at this time increased. The University of Mali (founded in 1993) offered pharmacists and medical doctors additional training options in medical biology. Cheikh Anta Diop University in Dakar offered training for senior laboratory technicians. In Burkina Faso, a three-year training programme for laboratory technicians started in 1985 at the *Ecole Nationale de Santé Publique*, while during the Sankara regime some nurses, including one key informant, were sent for laboratory medicine training in Cuba. Before 1985, most Burkinabé laboratory technicians were trained in Dakar, and some in Canada and France. The year 1999 saw the start of the *licence professionnelle option analyses* at the University of Ouagadougou.

Insufficient local training opportunities persisted for assistant and specialisation levels. Also, no formal training existed for laboratory assistants and nurses trained on-the-job, who still formed a large portion of laboratory personnel.^27^ The saying went: ‘You are a laboratory worker and you die a laboratory worker’. Due to the lack of laboratory training and career opportunities, many nurses left laboratory work to pursue further nursing training, although some nurses among our key informants stayed because they liked the work. A clinical biology university professor explained that because there was a lack of advanced specialisation training in medical biology or biochemistry in Senegal, medical doctors such as himself had to go for further studies abroad, mainly to France.

#### (In)visibility of laboratories in national policies and programmes

The first national Demographic and Health Surveys were conducted in the mid-1980s. National health plans focused on equity in service access through primary healthcare, the reduction of infant, child and maternal mortality, and family planning.^28^ These Demographic and Health Survey reports and national plans made little reference to laboratories. Only in 1999 did the Malian public health sector set up a referral system for laboratory tests: from first-level *Centre de Santé de Référence* to second-level regional hospitals to third-level university hospitals.^24^

No national funds were dedicated to laboratories, and therefore laboratory operations relied on budgetary allocations from health facility management. In those periods of economic austerity, the management of health facilities struggled to maintain all services, including laboratory services. Limited budgets and the consequently erratic reagent availability made it difficult for laboratories to function well.^28^ Biological pharmacists among the key informants explained that it was demotivating and boring to work in laboratories with so little support and rudimentary equipment when from their training they knew that the technology was more advanced in Europe. However, some doctors in charge of health facilities took the initiative to strengthen their laboratories. A director at the Reproductive Health Directorate Senegal recounted how when he became the medical director of a district health centre (1995–1998), the laboratory gained reference in the area because he asked a French friend to help him equip the laboratory with full blood count and glycaemia machines.

Laboratories became more visible in Senegal in 1990 as part of the *Direction de la Pharmacie et des medicaments avec volet Laboratoires*,^29^ and in Burkina Faso in 1993, when they gained a place in the *Direction Générale de la Pharmacies, des Medicaments Traditionels et des Laboratoires*. A current health inspector reasoned that the Burkinabé MoH realised the need to coordinate and supervise laboratory activities because the number of laboratories had increased.

#### Disease and donors

Many informants pointed out that the rapid development of laboratories from the 1990s onwards was mainly linked to AIDS control programmes which, compared to tuberculosis and malaria control programmes, required more than reagents and microscopes. Once HIV cases were discovered in the mid-1980s, national AIDS programmes were set up with large international donor support to gather epidemiological data. Until the mid-1990s, HIV testing was centralised – either the suspected HIV-positive individuals had to go to central laboratories or the laboratory staff went to the regions. A director of the Senegalese *Direction des Laboratoires* remembered that in cases of suspected HIV, laboratory staff had to travel to the regions to collect the blood samples and bring them to Dakar for analysis.

HIV serology and immunology tests were gradually decentralised to the regional hospital level. Donors supported regional hospitals and selected district health centre laboratories with equipment and supplies and by training laboratory staff – often assistants – to execute specific tests. The president of the Burkinabé association of technicians explained that in 1999 the entrance qualifications for laboratory technician training at the *Ecole Nationale de Santé Publique* were raised from a middle-school exam to *baccalauréat*-level (final exam of secondary school) because the sophisticated equipment and more complicated techniques required more highly trained laboratory personnel.

Two important drivers for laboratory development were the 1996 World Health Organization (WHO) meeting in Ouagadougou and the 1998 meeting in Bamako on epidemic preparedness and the vital role of laboratories. Ministry of Health representatives from 16 West African countries participated. Key informants who attended these meetings remembered that the WHO stressed laboratory development, training of laboratory staff for early diagnosis of epidemic diseases, and setting up of national laboratory networks. Some few years later, all MoHs agreed to support medical laboratories to prevent and fight epidemic diseases.

### Emergence of global health (late 1990s–present)

#### Increasing but suboptimal laboratory services

In all three countries, more public regional hospitals and health centres were built from the late 1990s onwards, which included buildings or rooms dedicated to laboratory services.^30^ Referral laboratories were connected to university hospitals and research laboratories were established for specific diseases. Compared to Mali and Burkina Faso, Senegal had fewer private stand-alone laboratories. In 2012, while Senegal had only 6, Burkina Faso had 80.^31,32,33^

With the arrival of more equipment and machines, the array of tests that laboratories could process increased.^34,35^ The head of the Senegalese AIDS control programme and the head of the WHO HIV programme in Ouagadougou explained that technological developments enabled further roll-out of the Prevention of Mother to Child (HIV) Transmission (PMTCT) programme. Around 2006, all health centre laboratories could perform HIV confirmation diagnostic tests. A Burkinabé pharmacist noted doctors began to increasingly use laboratory services when they observed a significant increase in the number of laboratories, tests and more qualified laboratory personnel. Other key informants observed that some younger doctors even overused the laboratory, with one expressing:

If the person [clinician] knows he can have a haemoglobin count, he does not bother to check the eyes or tongue of the patient, because that would take him more time than to write a test request. (Key informant 47, female, interview date 04 May 2015, Ouagadougou)

Medical laboratories exist from the health centre level. However, many (public) laboratories are often substandard. They have inadequate qualified human resources, often face stock-outs of rapid tests, encounter recurrent machine maintenance problems and non-availability of supplies and reagents, and have inadequate laboratory space. The latter was particularly true in Mali. A Malian biological pharmacist described how a health centre would find a laboratory space: ‘Here there is a spare storeroom that only has to be transformed to be a laboratory’ (Key informant 28, male, interview date 14 August 2015, Bamako). Laboratory staff worried that working in a non-dedicated room compromises safety and biosecurity. For instance, a laboratory technician in a Malian health centre complained that laboratory training was not practicable in these settings. For instance little space means that equipment is sometimes placed too close to the wall, or on top of each other.

Some health centre laboratories are still headed by inadequately trained staff. Mali has a shortage of clinical biologists due to the lack of training opportunities. In Burkina Faso, biological pharmacists are available, but many opt to work in private pharmacies, so they are ‘lost for the [public] laboratory’. In Senegal, the bottleneck is the result of an insufficient budget to recruit the many required trained laboratory technicians.

#### Improved organisation

The three countries’ MoHs realised the need to prepare and enable health facility laboratories to quickly respond to epidemic threats and to be less dependent on specialised research laboratories. National plans began to increasingly include laboratory services: national laboratory networks, directorates and sub-directorates. Interestingly, an outbreak of meningitis in Burkina Faso in 2002 led directly to the installation of the *Direction des Laboratoires* as one of the four technical sub-directorates of the *Direction Générale de la Pharmacie, du Médicament et des Laboratoires* (décret n°2011/PRES/PM/MS). Two key informants who were involved in this recounted what had made the MoH realise the importance of laboratory service coordination. During the outbreak, the country’s meningitis research laboratory had identified the outbreak virus to be a different strain (W135) previously unknown in the country, brought by pilgrims from Mecca. However, this discovery was not communicated to the MoH, leading to a wasteful high-cost countrywide meningitis A vaccination campaign.

In Mali, the national network of laboratories was created in 2004, consisting of specialised laboratories, hospital and *Centre de Santé de Référence* (district) laboratories, a few CSCom (sub-district) laboratories and private laboratories. In 2011, the *Division des Laboratoires* was added to the directorate of pharmacy and medicines. In Senegal, the laboratory was in 2002 included in the *Direction de la Pharmacie et des Laboratoires* and in February 2005 the national network of laboratories was officialised. This network extended to laboratories at the health centre level.^36^ Senegal created a *Direction des Laboratories* in 2012.^31^ Its director narrated the 10-year process of its birth: he advocated for its creation in 2002, and the then minister of health had been in favour; however, she was replaced and the new minister was less interested. When the former health minister was reinstated in 2012, the directorate was created.

A public health medical doctor at WHO Dakar explained that the advantage of being a stand-alone directorate is visibility at the ministry level, which goes with resource allocations. The problem of not having a budget for the sub-directorate in Burkina Faso was explicated by its former director: ‘I was appointed director without materials, without anything. It is with my car that I visited all the laboratories in the country [for quality assessment]’ (Key informant 30, male, interview date 04 May 2015, Ouagadougou). The director of the Division of Laboratories in Mali noted that the division still struggles to function well because of a lack of resources for personnel.

The national laboratory networks, the (sub-)directorate and division depend heavily on donor funds for their outreach activities such as general supervision and quality control. However, these donors mainly support disease-focused programmes and outreach stops when the programme ends. For example, laboratory supervision activities in Mali no longer take place since money from the Global Fund to fight AIDS, tuberculosis and malaria stopped.

To improve organisation and monitoring, the *Réseau d’Afrique de l’Ouest des Laboratoire* was created in 2009. RESAOLAB is a regional laboratory network that includes Senegal, Burkina Faso and Mali, domiciled in Bamako funded by *Agence Française de Développement* and *Fondation Mérieux*.

#### National laboratory policies and plans

The WHO guided the three MoHs in developing national laboratory policies and strategic plans. The first step was to conduct a countrywide inventory and evaluation of the status of laboratories. The United States Centres for Disease Control and Prevention (CDC) funded this exercise in Mali in 2012, and the Malian national laboratory policy was accepted in the same year. In Burkina Faso, the national laboratory policy was endorsed by the government in 2007, including the first five-year strategic plan.^37^ Senegal has no approved plan yet. The laboratory became more integrated into the health system through the specific mention of laboratory testing in management guidelines for certain conditions. For example, in Senegal and Mali, the antenatal care guidelines identify laboratory tests that all pregnant women should receive.^38,39,40^

#### Increased training opportunities

In-country training opportunities for all levels of laboratory personnel have increased since the late 1990s, and medical staff could specialise in laboratory sciences. Public and some private schools (the latter more prevalent in Senegal) were opened and existing schools or universities offered new curricula. Since 2007, laboratory technicians can upgrade their qualification through the Bachelor in Applied Medical Biology (*Biologie Médicale Appliquée – BAMS*) in Bamako, organised in collaboration with the *Centre d’Infectiologie Charles Mérieux, Fondation Mérieux*, and *Université Catholique de Lyon*. In 2010, a nine-month training programme started at *Ecole Nationale de Santé Publique*, Burkina Faso, for lower-trained technicians to upgrade to the same level as the later-trained *baccalauréat*-level technicians. The association of laboratory technicians had pushed for this training, and as of 2015 nearly all had been trained. Specialised training in laboratory sciences for medical doctors and pharmacists (*Diplôme d’Études Spécialisées de Biologie Clinique* [DES BC]) is offered in Ouagadougou (since 2004), Dakar (since 2008) and Bamako (since 2013). A *Diplôme d’Études Spécialisées* lecturer in Dakar explained that these regional training opportunities are cost-efficient because students no longer need to go overseas for *Diplôme d’Études Spécialisées* training, as his generation had to do. Since 2010, *Réseau d’Afrique de l’Ouest des Laboratoires* organises refresher training in dedicated institutes in all three countries for biologists and senior technicians working in laboratories, including modules on quality control, biosecurity and biosafety, and health information systems.

A general problem reported for many laboratory training programmes is the scarcity of equipment and supplies for hands-on practice. A lecturer in Dakar explained how lecturers have to buy equipment and reagents for the students’ practical work because since 2008 or 2009 the university does not receive government funding for research: ‘We do not even have a bottle of reagent for the practical work. The state has no money’ (Key informant 60, male, interview date 10 March 2015, Dakar). To tackle this problem, Senegalese university and training school laboratories have been allowed to generate money by offering paid services to the public.

#### Donor support focusing on specific diseases

Donors have continued to support specific laboratories with training and equipment for the disease they focus on. HIV programmes have greatly increased the availability and level of laboratory services for diagnosis and treatment follow-up. International donors, including the United States CDC, the Bill and Melinda Gates Foundation, the Clinton Health Access Initiative, the World Bank and the Global Fund, have financially supported or provided equipment, supplies and staff training. The Global Fund and World Bank financially supported infrastructure, surveillance, and supervision of HIV programmes. The United States CDC gave technical and financial support for sentinel site surveillance of HIV and syphilis among pregnant women, while providing quality controls for the testing services. The WHO provided financial and technical support for laboratory services to fight (other) epidemic-threat diseases, focusing on bacterial meningitis, bacterial diarrhoeal diseases, yellow fever, measles and rubella. In 2004, the WHO conducted training on the *Stratégie de surveillance integrée de la maladie et la riposte* in Ouagadougou, which was attended by several key informants. The training covered, among other things, epidemiology, security, quality assurance and laboratory systems development. In Burkina Faso, this training led to the National External Quality Assessment programme in 2006, supervision of laboratory systems and several training programmes in biosecurity.^41^ The WHO published a document on laboratory development^42^ that all countries ratified – the key informant at the WHO Burkina Faso office was one of the authors.

The West African Ebola epidemic of 2013–2016 was a wake-up call for donors to support laboratories and for governments to establish national research laboratories. The INRSP already existed in Mali, and the institute’s ex-director and a current lecturer both recounted how donors had supported the INRSP with equipment that enabled it to perform Ebola diagnostic tests on regional samples. These key informants believed that this timely and efficient action contributed to the containment of Ebola in Mali.

Many key informants criticised donor support for its focus on specific diseases. The former head of several Senegalese MoH directorates (2004–2012) complained that donors for AIDS and malaria programmes only support selected laboratories with equipment and training. Two biotechnologists active in the Malian national association of laboratory technicians highlighted the lack of long-term vision and soundness of donor support during the Ebola outbreak when just a few technicians were trained and general biosecurity was only maintained as long as Ebola was a threat. A Burkinabé pharmacist recounted how a donor had given 10 microscopes – even though the (vertical) programme only needed three – but had not allowed the extra seven microscopes to be used for other services. Informants also criticised the fact that some donors imported their machines, not considering what was already available. For example, the head of the Directorate for Sexual and Reproductive Health in Burkina Faso recounted how, as a medical doctor in the region, she experienced that the laboratory equipment given by donors differed from what is normally used in the country. For instance, they received CD4 count machines that used different reagents and could be maintained or repaired by only one person in West Africa when the machines were out of commission for three months.

*Réseau d’Afrique de l’Ouest des Laboratoires* is one of the few donor-funded programmes focusing on the laboratory *system*. It supports the training of different levels of laboratory staff, the renovation and equipment of laboratories, and the equipment of training centres.^43^ Involved key informants were very positive about the contributions of *Réseau d’Afrique de l’Ouest des Laboratoires*. Some other programmes and funds also address laboratory systems, including the Global Health Security Agenda (launched 2014), the Regional Disease Surveillance Systems Enhancement II project and the West African Health Organisation which conducts large laboratory system strengthening through the World Bank funding.

## Discussion

Until the late 1990s, there were few investments in the expansion of laboratory capacity, partly as a result of economic austerity that affected the overall health sector, but also because laboratories were not considered a priority. From the 1990s onwards, the development of laboratories was mainly influenced by the emergence of potential pandemic diseases that require laboratory confirmation for treatment and control. New technology has led to the expansion of laboratory services. However, operating a laboratory (including machines and equipment) depends on the availability of amenities such as electricity and running water. At the time of this report, unavailable and erratic public amenities challenge proper laboratory functioning. Not only do power cuts hinder testing, power fluctuations also cause equipment breakdown.

The MoHs in these economically constrained countries have had and still do have to operate with limited public finances, relying heavily on external financial aid for the running of public health services. Donors have therefore played the most important role in how laboratories have been developmentally geared towards specific diseases; national policymakers did not take a leading role. Dependence on donors puts the MoHs in a weak position in terms of setting priorities for laboratory development and research. Donor support for specific diseases did and might not strengthen the laboratory system for the diagnosis and follow-up of all health conditions.

An unexpected finding was the big influence of ‘laboratory champions’: persons committed to laboratories, who have a ‘vision’ and ‘fight’ for the development of the national laboratory system. They are individuals who got ‘the laboratory virus’, as the director of the Senegalese Directorate of Laboratories aptly described them. These champions included biologists and pharmacists who lobbied national ministers of health and donors to set up research laboratories, national health ministers and laboratory technicians active in their associations. These champions were often constrained in putting their vision into practice by the larger national context of unsupportive political leadership, general poverty and the lack of basic infrastructure and amenities.

### Recommendations

Drawing lessons from the study findings, the following recommendations for laboratory-strengthening programmes are directed at political leaders, MoHs, health facilities and donors:

- Have a stand-alone directorate of laboratories with a dedicated national budget, as per the recommendation of the 2008 Maputo Declaration.^44^ The WHO Regional Office for Africa, the African Society for Laboratory Medicine and the Africa CDC should continue to remind and support national policymakers who signed the declaration. National policymakers should dedicate more national budget to laboratory development and give the national directorate the mandate to coordinate and guide donor involvement in public and private laboratories at all levels.
- Prioritise continuous professional development opportunities for laboratory personnel at all levels, including laboratory assistants trained on-the-job. Professional councils and associations, and the African Society for Laboratory Medicine, should play a role in regulating the profession while the national leadership supports with sufficient funding.
- National public health laboratories should be established – this is one of the goals of the Africa CDC, supported by the African Society for Laboratory Medicine – and should have political influence at the MoH to set national research priorities.
- Health facility management committees should establish a dedicated budget for the laboratory, reasoning that the laboratory is a source of direct income and needs to function optimally. As the director of a Senegalese regional hospital expressed: ‘That will make the facility function. The laboratory and the radiology are the “lungs” of the health facility’ (Key informant 65, male, interview date 16 March 2015, Kaolack).

### Limitations

The authors acknowledge that personal accounts do not constitute hard, objective data, as is the norm in the medical sector, including laboratory medicine. The authors are also aware that respondents may have constructed this history of, and recommendations for, laboratory development to suit their own or their organisations’ interests. Nevertheless, by triangulating literature and accounts of key informants from three countries and different health professions and levels, the authors opine that this oral history presents a true reflection of the development of medical laboratories in francophone West Africa.

### Conclusion

This unique article on the long-term historical developments of the laboratory sector in francophone West Africa demonstrates that by collecting and recording the experiences of people who lived through and have been actors in laboratory developments, a history that would have been otherwise forgotten has been constructed. Many of the historical laboratory sector challenges still exist and will not be surmounted without national leaders prioritising the alleviation of national poverty and the development of basic infrastructure. As exemplified by the champions described in this article, national policymakers must be abreast of their population’s disease burdens and needs and play the important leadership role to donors ensuring donor programmes match the populace’s healthcare or laboratory needs, consequently advancing their population’s healthcare. Ministries of Health should see laboratories as an integral part of the health system, and not simply as part of vertical disease programmes. The retired director of INRSP in Mali aptly reiterated: ‘The laboratory is the brain of the health system’ (Key informant 24, male, interview date 13 August 2015, Bamako).
